# Patterns of Fourier-transform infrared estimated milk constituents in early lactation Holstein cows on a single New York State dairy

**DOI:** 10.3168/jds.2022-22588

**Published:** 2023-02-22

**Authors:** K. R. Callero, E. M. Teplitz, D. M. Barbano, C. R. Seely, J. A. Seminara, I. R. Frost, H. A. McCray, R. M. Martinez, A. M. Reid, J. A. A. McArt

**Affiliations:** 1 Department of Population Medicine and Diagnostic Sciences, College of Veterinary Medicine, Cornell University, Ithaca, NY 14853; 2 Department of Public and Ecosystem Health, College of Veterinary Medicine, Cornell University, Ithaca, NY 14853; 3 Department of Food Science, College of Agriculture and Life Sciences, Cornell University, Ithaca, NY 14853; 4 College of Agriculture and Life Science, Cornell University, Ithaca, NY 14853; 5 College of Veterinary Medicine, Cornell University, Ithaca, NY 14853; 6 College of Arts and Sciences, Cornell University, Ithaca, NY 14853

**Keywords:** milk constituents, parity, fatty acids, Fourier-transform infrared spectroscopy

## Abstract

Cows undergo immense physiological stress to produce milk during early lactation. Monitoring early lactation milk through Fourier-transform infrared (FTIR) spectroscopy might offer an understanding of which cows transition successfully. Daily patterns of milk constituents in early lactation have yet to be reported continuously, and the study objective was to initially describe these patterns for cows of varying parity groups from 3 through 10 d postpartum, piloted on a single dairy. We enrolled 1,024 Holstein cows from a commercial dairy farm in Cayuga County, New York, in an observational study, with a total of 306 parity 1 cows, 274 parity 2 cows, and 444 parity ≥3 cows. Cows were sampled once daily, Monday through Friday, via proportional milk samplers, and milk was stored at 4°C until analysis using FTIR. Estimated constituents included anhydrous lactose, true protein, and fat (g/100 g of milk); relative % (rel%) of total fatty acids (FA) and concentration (g/100 g of milk) of de novo, mixed, and preformed FA; individual fatty acids C16:0, C18:0, and C18:1 *cis*-9 (g/100 g of milk); milk urea nitrogen (MUN; mg/100 g of milk); and milk acetone (mACE), milk β-hydroxybutyrate (mBHB), and milk-predicted blood nonesterified fatty acids (mpbNEFA) (all expressed in mmol/L). Differences between parity groups were assessed using repeated-measures ANOVA. Milk yield per milking differed over time between 3 and 10 DIM and averaged 8.7, 13.3, and 13.3 kg for parity 1, 2, and ≥3 cows, respectively. Parity differences were found for % anhydrous lactose, % fat, and preformed FA (g/100 g of milk). Parity differed across DIM for % true protein, de novo FA (rel% and g/100 g of milk), mixed FA (rel% and g/100 g of milk), preformed FA rel%, C16:0, C18:0, C18:1 *cis*-9, MUN, mACE, mBHB, and mpbNEFA. Parity 1 cows had less true protein and greater fat percentages than parity 2 and ≥3 cows (% true protein: 3.52, 3.76, 3.81; % fat: 5.55, 4.69, 4.95, for parity 1, 2, ≥3, respectively). De novo and mixed FA rel% were reduced and preformed FA rel% were increased in primiparous compared with parity 2 and ≥3 cows. The increase in preformed FA rel% in primiparous cows agreed with milk markers of energy deficit, such that mpbNEFA, mBHB, and mACE were greatest in parity 1 cows followed by parity ≥3 cows, with parity 2 cows having the lowest concentrations. When measuring milk constituents with FTIR, these results suggest it is critical to account for parity for the majority of estimated milk constituents. We acknowledge the limitation that this study was conducted on a single farm; however, if FTIR technology is to be used as a method of identifying cows maladapted to lactation, understanding variations in early lactation milk constituents is a crucial first step in the practical adoption of this technology.

## INTRODUCTION

Some of the most pertinent issues affecting cow health are the energy, protein, and macromineral deficits that occur directly after parturition. Cows are unable to consume enough feed to meet strenuous demands during the transition period, leaving them in an extended state of energy deficit, and one of the main energy draws is the onset of lactogenesis (Bauman and Currie, 1980; Herdt, 2000). The inability to compensate for these changing metabolic demands increases a cow’s risk of diseases, some of which may be subclinical in nature. Because many nutrients are being redirected for use in the udder, milk analysis might provide further insight into a cow’s homeorhetic adaptation to lactation.

Development and research in Fourier-transform mid-infrared analysis (**FTIR**) technology has allowed us to investigate milk as a health indicator for early lactation cows (McParland and Berry, 2016). Early reports of FTIR predictions centered on its use as a screening method for elevated milk acetone (**mACE**), milk BHB **(mBHB**), and other milk biomarkers of health (Hansen, 1999; Heuer et al., 2001; de Roos et al., 2007). Additional research has described the relationship of milk constituent patterns in early lactation (Santschi et al., 2016; Tatone et al., 2017; Kowalski et al., 2021) and investigated the relationship of these patterns with disease (Bach et al., 2019). Although Santschi et al. (2016), Tatone et al. (2017), and Kowalski et al. (2021) routinely examined milk throughout early lactation in a large population of cows on multiple dairies, they observed the changes in constituent patterns from month to month. Monthly milk testing can be useful on the herd level but does not offer any insight into an individual cow’s milk progression. Bach et al. (2019) initiated the step into more frequent sampling with 2 tests a week for a total of 4 recorded milk tests. From these data, it became clear that milk constituents were associated with disease and needed to be further investigated. It must be noted that the ability to use milk to assess health is nuanced, as milk composition changes depending on the frequency and time of day it is taken, which can lead to multiple confounding factors (Seely et al., 2022). In addition, a review by De Marchi et al. (2014) noted the importance of taking the stage of lactation, breed, diet, and herd location into account when assessing FTIR-estimated milk components.

While observing milk constituent patterns, some parity effects have been discovered. The best-known difference is that primiparous cows produce less milk than multiparous cows (Beauchemin and Rode, 1994). Nielsen et al. (2005) noted that, in addition to milk yield, individual-cow variables such as parity and DIM were important factors to consider in models when identifying the risk of ketosis using mBHB. Parity effects on milk constituents, taken from monthly milk tests, have been found for mACE, mBHB, and de novo fatty acids (**FA**) in several studies (e.g., Santschi et al., 2016; Tatone et al., 2017; Mäntysaari et al., 2022). Among the findings in these studies, Kowalski et al. (2021) reported that mACE values were greater in parity 1 and ≥3 cows than in parity 2 cows, and mBHB was greatest in parity ≥3 cows compared with parity 1 and 2 cows. De novo FA concentrations were less in parity 1 cows compared with parity ≥2 cows (Van et al., 2020). However, there is currently a paucity of information investigating the daily change of milk constituents in early lactation. Thus, our study objectives were to initially describe milk constituent patterns for cows of differing parities sampled daily from 3 through 10 DIM and investigate differences in these patterns between parity groups on a single dairy farm. We hypothesized that parity would be an important factor in describing patterns of milk constituents within the first 10 DIM.

## MATERIALS AND METHODS

### Study Design and Approval

We conducted a prospective observational cohort study that was analyzed and written following the STROBE-Vet guidelines for strengthening the reporting of observational studies in veterinary epidemiology. Due to the descriptive nature of the study and lack of previous longitudinal data for repeated milk sampling of individual cows, we did not calculate a sample size a priori. The research application for the project was reviewed by the Cornell University Institutional Care and Use Committee office. As there was no animal contact or manipulation in the trial, the project did not meet the regulatory Cornell Policy 1.4 description for use of animals for research, teaching, or testing. Therefore, the project was granted an exemption from Institutional Care and Use Committee review. The project was then evaluated and approved by the Cornell University Veterinary Clinical Studies Committee following an ethical and scientific review (CUVCSC Protocol ID# 062420–05). The participating farm signed a consent form allowing us to conduct the study.

### Study Population

We collected data from 1,166 early lactation Holstein cows on a single dairy farm in Cayuga County, New York, from June through August 2021. The farm was selected for its working relationship and previous successful participation in studies conducted through the Cornell Ambulatory and Production Medicine Clinic (Ithaca, NY). The farm milked approximately 4,400 cows thrice daily in a 100-stall rotary parlor (DeLaval) and averaged 45.6 kg of milk (4.0% fat and 3.1% protein) per day per cow during the study period, with parity 1 cows averaging 39.5 kg/d, parity 2 cows averaging 47.9 kg/d, and parity ≥3 cows averaging 49.5 kg/d. Cows were housed in freestall barns on recycled manure bedding. After parturition, cows were milked once in the maternity area within 8 h of calving and then moved to an early lactation pen, which commingled primiparous and multiparous cows. Cows were fed once daily at 0745 h and milked at 0230, 1030, and 1830 h. The ingredients and formulated nutrient composition of the early lactation diet, calculated as an average from 5 different diet estimation timepoints throughout the study period, are included in Table 1. Cows exited the early lactation pen between 7 and 21 DIM at the discretion of the farm manager, based on parity, daily milk yield, and pen stocking density. Cows were eligible for inclusion in the study if they were in the early lactation pen on any day during the sampling period.

### Animal Sampling

We collected proportional milk samples from all cows in the early lactation pen once daily, Monday through Friday, at the mid-morning milking at 1030 h, with an average of 4 ± 2 samples collected per cow. Cows were automatically identified upon entry to the milking parlor by a radiofrequency identification detection tag and visually confirmed by the research team. As the cows were milked, their identification and stall numbers were dictated into a voice app; recorded milking order and identification numbers were confirmed with computer records from the parlor software (DelPro; DeLaval).

Milk samplers (DeLaval Fat Sampler MM25–27 BC; DeLaval) were used to collect proportional milk samples from every cow. Once a cow finished milking, the sample cups were removed from the samplers, inverted 3 times, and then poured into 60-mL plastic vials without preservatives (Aptar CSP Technologies). Each vial was prelabeled in numeric order corresponding to the order in which cows were milked. Milk samples were immediately placed in an ice bath at 4°C and transported to the Barbano Laboratory in the Department of Food Science at Cornell University (Ithaca, NY) for FTIR milk analysis.

We extracted farm-diagnosed disease outcome data of milk fever, mastitis, metritis, ketosis, displaced abomasum, and culling from the farm software (Dairy-Comp 305; Valley Agricultural Software). Milk fever was diagnosed as a recumbent cow between 0 and 2 DIM with cold ears. Cows were flagged for physical examination by the computer system when their milk yield deviated from an expected calculated weight, and trained farm employee exam findings led to diagnoses of ketosis, metritis, or displaced abomasum. Diagnosis of metritis occurred if cows were systemically ill with a rectal temperature ≥39.5°C and reddish-brown uterine discharge. Ketotic cows had blood BHB concentrations ≥1.2 mmol/L on a cow-side BHB meter blood test (PrecisionXtra; Abbott). A diagnosis of displaced abomasum occurred if a “ping” sound was heard during simultaneous auscultation and percussion of the cow in a line from the tuber coxae to the olecranon. Mastitis was diagnosed by farm employees in the parlor when they detected a hot swollen quarter, presence of abnormal milk, or both. Once diagnosed, cows were treated following farm protocols developed by the farm veterinarian. Farm employees were blinded to study details.

### Milk Composition Analysis

Proportional milk samples were analyzed in the Barbano Laboratory at the Department of Food Science at Cornell University (Ithaca, NY). On average, milk samples were analyzed within 0.6 ± 0.7 d of collection. An FTIR spectrophotometer (Lactoscope model CombiScone FT600, Delta Instruments) was used to analyze the contents of milk fat, true protein, and anhydrous lactose. As previously described by Kaylegian et al. (2009), prediction models utilized were the optimized basic model filter wavelengths, which were then validated by AOAC official validated chemical reference methods as described in Wojciechowski et al. (2016).

Woolpert et al. (2016) describes the partial least squares (**PLS**) prediction models and version numbers that were used to measure the concentration (g/100 g of milk) for de novo (C4 to C14), mixed (C16, C16:1, and C17), and preformed (≥C18) FA. Relative percentages (**rel%**) of the 3 FA groups were calculated by taking the concentration of each FA group and dividing it by the total milk FA concentration (both of which are reported in g/100 g of milk). To calibrate the milk FA parameters, gas-liquid chromatography reference chemistry was used as described previously (Wojciechowski and Barbano, 2016). The 14-sample set used to calibrate the main milk constituents was also used as the milk FA calibration set. Calibration concentration ranges, estimated yields, and PLS modeling parameters and version numbers for C16:0, C18:0, and *cis-*9 C18:1 were as stated in Seely et al. (2022). A new 14-sample milk calibration set was produced and used to adjust the slope and intercept of each component once every 4 wk. The set was run on the instrument each week on Monday morning to check mean differences between the instrument and reference values for each parameter to ensure the mean difference in g/100 g of milk was <0.01%. The typical standard deviation of the differences among the reference chemistry and instrument-predicted values for the 14-sample calibration set was <0.015 g/100 g of milk for each parameter. Milk urea nitrogen (mg/100 g of milk) and milk-predicted blood nonesterified fatty acids (**mpbNEFA**), mBHB, and mACE (expressed as mmol/L) were measured by FTIR using a PLS model developed by Delta Instruments using parameter version numbers #502, #1603, #1601, #1602, respectively (Bach et al., 2019, 2021) and calibrated using reference chemistry values from an enzymatic MUN assay (Portnoy et al., 2021).

### Statistical Analysis

Initially, the data set was processed to remove any samples that did not fall within the selected 3 to 10 DIM range. Samples from cows at 0 through 2 DIM were excluded to retain the accuracy of the models, given that the PLS models were created with spectra starting at 3 DIM. Any cows not of the Holstein breed were excluded. To ensure the data set was representative of commercial dairies, only milk samples that were taken after a cow was diagnosed with a disease(s) listed above were removed from the data set. Subsequently, all daily milk data in which the sum of de novo, mixed, and preformed FA (g/100 g of milk) was >99% of the fat concentration (g/100 g of milk) was removed. Data within the 98 to 99% range that also had negative milk-predicted BHB values were removed. All samples with an FA sum <97% were retained. The majority of the removed samples were already excluded because they were collected from 0 to 2 DIM.

Data processing and graphs were generated using R software (version 4.1.2; https://www.r-project.org/). Raw mean milk yield per milking and corresponding 95% CI were calculated for each parity group (1, 2, and ≥3) at each DIM from 3 through 10.

To assess parity differences, changes in concentrations of milk constituents from 3 through 10 DIM were analyzed using generalized linear mixed models created through the MIXED procedure of SAS (v. 9.4; SAS Institute Inc.). Models included the random effect of cow and the fixed effects of parity group, milk yield per milking, DIM, and the interaction of parity group × DIM to assess changes over time as well as differences between parity groups for each of the following outcomes: anhydrous lactose, true protein, fat, MUN, de novo FA (g and rel%), mixed FA (g and rel%), preformed FA (g and rel%), C16:0 (g/100 g of milk), C18:0 (g/100 g of milk), *cis*-9 C18:1 (g/100 g of milk), mpb-NEFA, mBHB, and mACE. The outcome of milk yield was modeled in a similar manner without the inclusion of milk yield per milking as a fixed effect. Multiple measurements over time within the same cow were accounted for using DIM in the REPEATED statement. First-order autoregressive, Toeplitz, and unstructured covariance structures were tested, and the covariance structure with the lowest Akaike’s information criterion was selected. For all modeled constituents, this was a first-order autoregressive covariance structure, except for anhydrous lactose, which used a Toeplitz covariance structure. The fixed effect of milk yield was removed from models when *P* < 0.10. To improve the normality of residuals, the following transformations were made: log (fat), squared (anhydrous lactose), square root (true protein, MUN, mixed FA grams, and rel%, preformed FA rel%, C16:0, C18:0, *cis*-9 C18:1).

Means were considered statistically different when *P* ≤ 0.05 with marginal evidence for a statistical difference when 0.05 < *P* ≤ 0.10. When main effects and least squares means were different between groups, Tukey-Kramer studentized adjustments were used to account for multiple comparisons. Results are reported as the back-transformed least squares means and their associated 95% CI, which are displayed as shaded bands in the line plot figures.

## RESULTS

### Population Description and Descriptive Statistics

**Figure 1.**
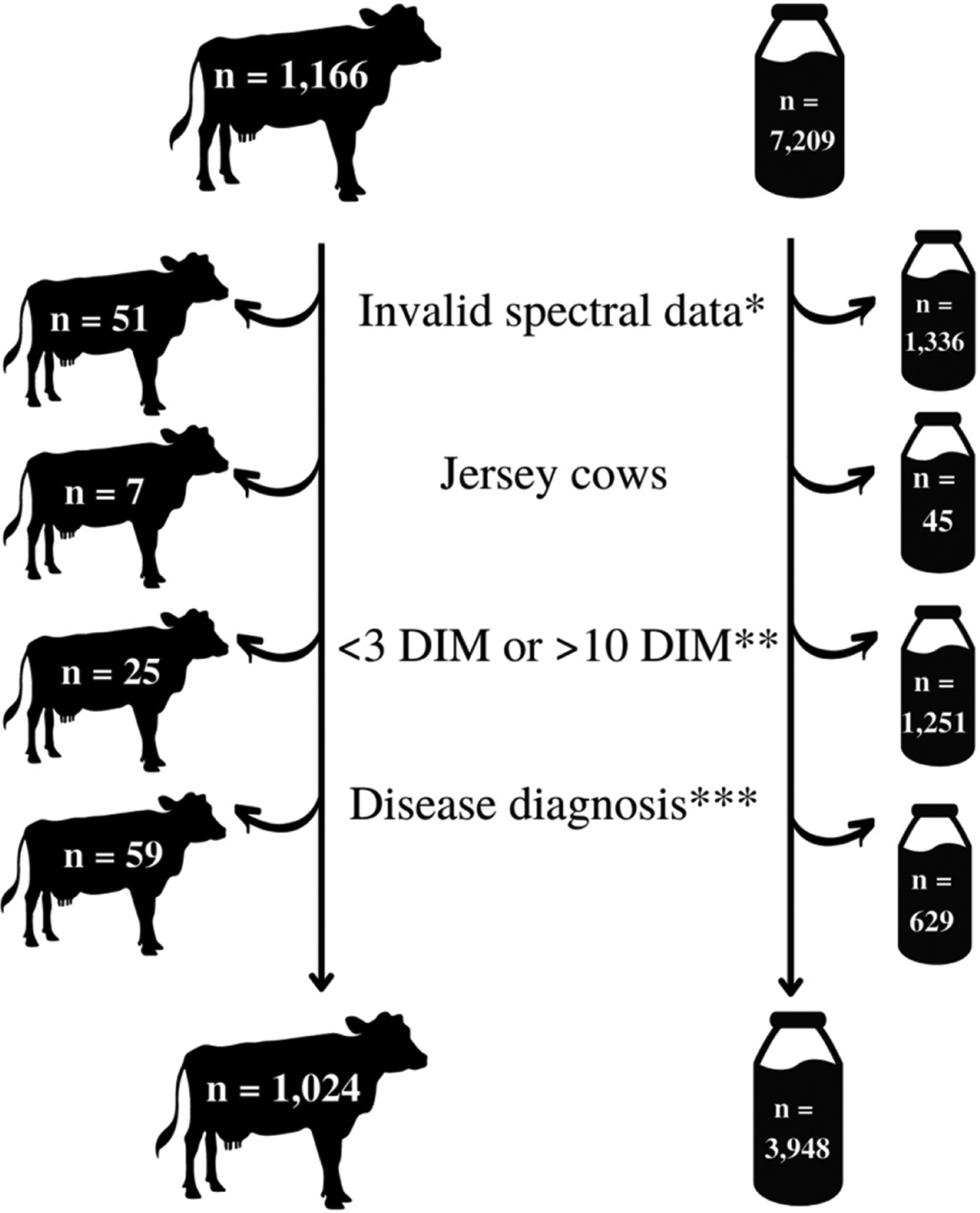
Diagram demonstrating the initial cow enrollment and milk sample numbers and the subsequent exclusionary reasons leading to a final enrollment of 1,024 Holstein cows and 3,948 milk samples from a commercial dairy farm in New York. *Overall, 70% of excluded spectral samples were from 0, 1, and 2 DIM; an additional 11% were excluded from 3 DIM. **Excluded cows: did not have any milk samples collected from early lactation pen between 3 and 10 DIM. Excluded milk samples: fell outside the noted range. ***Excluded cows: those diagnosed with a disease <3 DIM. Excluded milk samples: (1) all samples from cows excluded for having a disease <3 DIM, or (2) subsequent samples from cows diagnosed with a disease ≥3 DIM (e.g., a cow diagnosed with disease at 5 DIM had samples from 6 to 10 DIM excluded.

**Figure 2.**
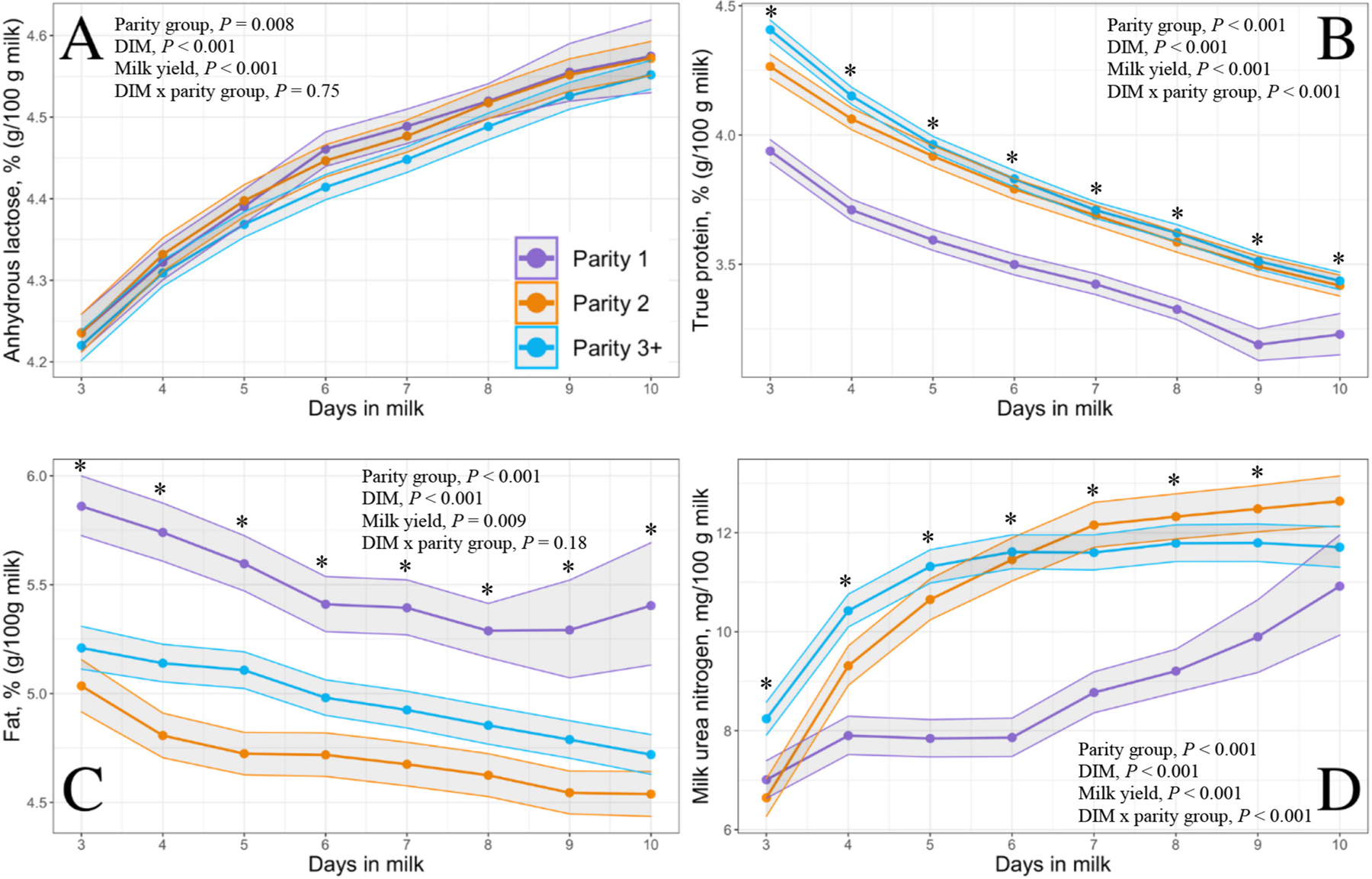
Final model LSM concentrations and 95% CI (shaded) of Fourier-transform infrared predicted (A) milk anhydrous lactose (g/100 g of milk), (B) true protein (g/100 g of milk), (C) fat (g/100 g of milk), and (D) milk urea nitrogen (mg/100 g of milk) from 3 through 10 DIM in 1,024 Holstein cows from a single dairy in New York. Parity groups are represented as parity 1 (purple; n = 306), parity 2 (orange; n = 274), and parity ≥3 (blue; n = 444). Asterisks represent a parity group difference at *P* < 0.05 at each respective DIM.

The 30 DIM incidences of disease and culling for cows enrolled during the study period are given in Table 2. Figure 1 illustrates the condensing of data after applying various exclusionary criteria. Of the 1,024 Holstein cows ultimately enrolled, 306 were parity 1, 274 were parity 2, and 444 were parity ≥3. Each DIM had the following number of samples: 3 DIM, n = 465; 4 DIM, n = 549; 5 DIM, n = 579; 6 DIM, n = 562; 7 DIM, n = 523; 8 DIM, n = 506; 9 DIM, n = 409; 10 DIM, n = 355. Raw means and 95% CI for milk yield per milking and each constituent of interest by parity group across 3 through 10 DIM are in depicted in Figures 2, to 5.

### Differences in Milk Constituents by Parity Group

**Figure 6.**
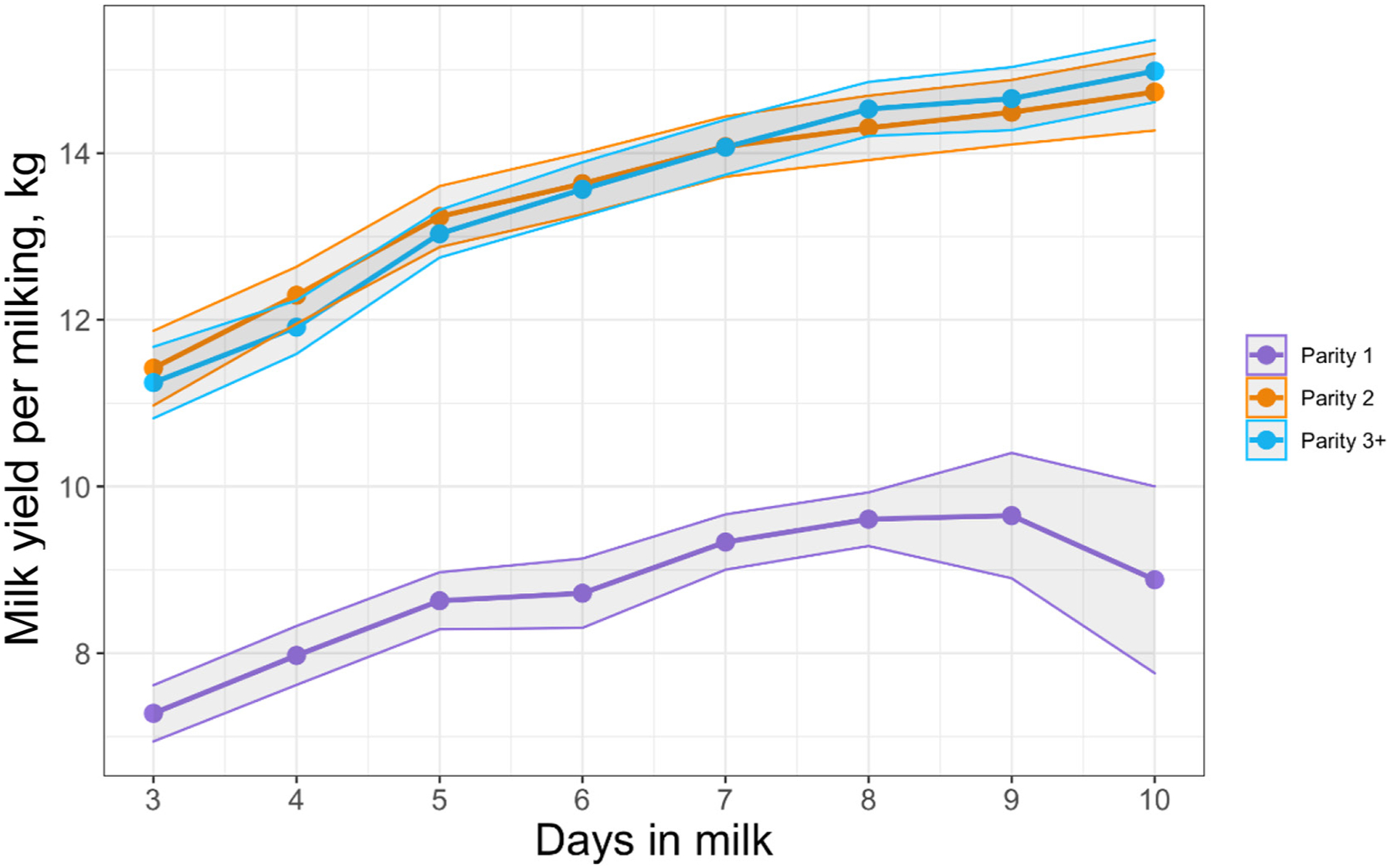
Raw mean and 95% CI (shaded) of milk yield per milking from 3 through 10 DIM in 1,024 Holstein cows from a single dairy in New York sampled once daily at the 1030 h milking. Parity groups are represented as parity 1 (purple; n = 306), parity 2 (orange; n = 274), and parity ≥3 (blue; n = 444).

Milk yield differed by parity group from 3 through 10 DIM with parity 1, parity 2, and parity ≥3 cows producing 8.7, 13.3, and 13.3 kg of milk per milking, respectively (*P* < 0.001; Figure 6). Modeled results of constituent outcomes comparing parity groups are summarized in Table 3. Parity differences were found for % anhydrous lactose (*P* = 0.008), % fat (*P* < 0.001), and preformed FA g/100 g of milk (*P* < 0.001). Parity differences across DIM were present for % true protein, de novo FA (rel% and g/100 g milk), mixed FA (rel% and g/100 g milk), preformed FA rel%, C16:0, C18:0, C18:1 *cis*-9, MUN, mACE, mBHB, and mpbNEFA (all *P* < 0.005). Parity 1 cows had decreased protein, MUN, and de novo and mixed FA rel% compared with parity 2 and ≥3 cows. Furthermore, parity 1 cows had elevated concentrations of fat % and preformed FA rel% compared with parity 2 and ≥3 cows (% fat: 5.55, 4.69, 4.95; preformed FA rel%: 53.54, 45.49, 47.09, respectively). Parity 1 cows had heightened preformed FA rel% concentrations, which correlated with the greatest concentrations of the relevant energy-deficit markers mpbNEFA, mBHB, and mACE compared with multiparous cows.

## DISCUSSION

Our long-term goal is to determine whether milk analysis can predict which cows adapt and thrive during the transition period and which struggle and need additional support. It is critical to identify cows maladapting to the onset of lactation to help prevent a reduction in milk-producing potential (Edwards and Tozer, 2004). By looking at daily milk constituent patterns, we hoped to establish a baseline for characterization of milk differences between parity groups in early lactation.

Of the 16 estimated milk constituents we investigated, only de novo FA (as g/100 g of milk) did not differ between parity groups from 3 to 10 DIM. Although a statistical difference was found in anhydrous lactose when evaluating the mean concentration from 3 to 10 DIM between parity groups, the difference was biologically nonmeaningful. Two estimated milk constituents, mixed FA (g/100 g of milk) and individual FA C16:0, only differed by parity group on a few of the individually investigated days, at 4 through 6 DIM and 4 to 5 DIM, respectively. Visualization of these 2 constituents showed that although there was a statistical difference at a small number of DIM, the biological relevance of this difference is likely not large and possibly a type I error. It is likely that in the study herd, parity group is not an important factor to consider when evaluating these constituents in early lactation.

However, the concentrations of 12 of the remaining estimated milk constituents, including true protein, fat, de novo FA rel%, mixed FA rel%, preformed FA (g/100 g of milk), preformed FA rel%, mpbNEFA, mACE, and the individual FA C18:0 and *cis*-9 C18:1, differed between parity groups every day between 3 and 10 DIM. Milk BHB differed between parity groups on every day except 6 DIM, which may represent a type II error rather than one of biological significance, and MUN differed between parity groups each day from 3 through 9 DIM but began to converge by 10 DIM. Thus, in the study herd, when evaluating differences between milk constituents in early lactation, it is important to not only identify parity group but also the changing patterns of each constituent over time from 3 to 10 DIM.

Interestingly, although milk yield per milking was a statistically significant factor in the majority of our outcomes of interest, it did not diminish the importance of parity group as a contributing factor to differences between milk constituent estimates. This suggests that milk yield does not create a “dilution factor” when evaluating milk constituents, which might be of relevance to herds without milk yield monitoring capability. Further studies on multiple farms are needed to ascertain whether milk yield is an important factor to consider when repeatedly estimating milk constituents or if it was merely a finding in this study herd.

Our study was designed to repeatedly sample individual cows to assess changes in milk constituents over time in early lactation between parity groups. Previous studies have often focused on a small number of early lactation cows, either directly measuring milk FA or via FTIR (Van Haelst et al., 2008; Jorjong et al., 2014, 2015; Mann et al., 2016) or monthly testing of larger populations to estimate milk constituents via FTIR (Santschi et al., 2016; Tatone et al., 2017; Kowalski et al., 2021). In addition, several of these studies only investigated multiparous cows or, while accounting for parity in their statistical analyses, did not report the importance of parity or discuss differences between groups. Although the differences in study design and sampling strategies between these studies makes direct comparisons difficult, a discussion of the general trends of parity differences and estimated milk constituents in early lactation is important.

The findings from our study support previous work showing comparable differences in milk constituents across parity groups through monthly milk testing regarding milk FA. Van et al. (2020) compared milk from primiparous (n = 39) and multiparous (n = 57) cows at 3 different timepoints from 7 to 150 DIM. Across the sampling period, their ≤50 DIM sample point was identified as having the lowest concentrations of de novo FA and highest concentrations of preformed FA, with primiparous cows having overall lower concentrations of de novo and mixed FA and greater concentrations of preformed FA than multiparous cows. However, it is unknown what specific timepoint (DIM) accounts for this difference because a DIM × parity interaction was not reported, nor were results stratified by DIM within parity. In 11 herds in Finland with 245 single-sampled cows, Mäntysaari et al. (2022) reported that primiparous cows had greater C18:1 *cis*-9 from 8 to 21 DIM than multiparous cows. Unfortunately, other studies investigating milk FA did so on a small number of cows, only multiparous cows, or sampled cows beyond the current study period of 3 to 10 DIM, which prevents appropriate comparisons (Van Haelst et al., 2008; Jorjong et al., 2014, 2015; Mann et al., 2016).

The rel% values of de novo FA were lowest for primiparous cows followed by parity ≥3 cows, with parity 2 cows having the highest concentration. De novo FA are synthesized in the mammary gland from substrates created in the rumen, such as acetate and butyrate, and thus de novo FA can be a good indicator of rumen health as well (Bauman and Griinari, 2003). Combined with our de novo FA results, the elevated preformed FA rel% found in primiparous cows may be indicative of greater adipose tissue mobilization compared with greater parity herd mates, even though the primiparous cows did not produce as much milk. It is possible that social dynamics within the early lactation pen reduced intake of parity 1 cows, thus increasing their energy deficit (DeVries et al., 2004).

Although the increase in preformed FA rel% in parity 1 cows within our study agreed with milk markers of energy deficit, these findings on milk markers of energy deficit and parity patterns differ from those of other studies. The greater mACE concentration patterns for parity 1 and ≥3 cows compared with parity 2 cows in Kowalski et al. (2021) were similar to those observed in our study; however, we found that the mACE concentrations were much lower than in the aforementioned study. In addition, our mBHB results conflicted with those of Kowalski et al. (2021), Santschi et al. (2016), and Tatone et al. (2017), as we found that parity 1 cows had the greatest mBHB concentrations followed by parity ≥3 and parity 2 cows. Kowalski et al. (2021) reported the same parity difference pattern in early lactation but observed that mBHB concentrations in parity 1 cows began to decline by 9 DIM, a finding that supported earlier results from Santschi et al. (2016) and Tatone et al. (2017), where the prevalence of mBHB ≥0.15 mmol/L increased from 5 through 10 DIM in multiparous cows but decreased in the same period in primiparous cows. However, overall, mBHB concentrations in the reported study were lower than those of the previously mentioned studies. In addition to a difference in daily versus monthly testing, it is worth noting the population difference between our single sample herd of 4,400 cows and prior studies: Santschi et al. (2016) reported on 498,310 samples from 4,242 herds in Quebec, Canada, with a mean herd size of 62 cows (range = 35 to 766); Tatone et al. (2017) reported on 165,749 cows from 3,042 herds in Ontario, Canada, with a mean herd size of 68 cows (range = 4 to 1,184); and Kowalski et al. (2021) reported on 3,867,390 cows from 21,300 herds in Poland, with a mean herd size of 38.7 cows (range = 1 to 1,356). Although no information was reported on the frequency at which the herds were milked or fed in the prior studies, the vast difference in mean herd size suggests there are likely management differences between these studies and the current study. This variation between study populations might contribute to the reported differences in mBHB and mACE. However, based on reported blood NEFA and BHB data (Duffield et al., 1998; McArt et al., 2013; Suthar et al., 2013), we expect multiparous cows to have greater mpbNEFA and mBHB given the greater milk production, but during the first 10 DIM, we found that parity 1 cows had greater mpbNEFA and mBHB than multiparous cows. It is entirely plausible that these differences are due to the management and nutritional factors of the researched dairy, and thus our findings may not be externally valid.

This study has some limitations. As we conducted the trial on only one farm, the findings are representative of cows on a single diet with a farm-specific management style. As seen in Castro et al. (2022), management practices such as feed pushing style, feed delivery frequency, bedding type, raking frequency, and barn design all contribute to milk FA concentrations recorded in cows. Grummer (1991) noted that milk fat can be altered through the diet (e.g., feeding a low roughage diet to increase levels of C18:1), and milking frequency and ration energy levels also change milk composition (Wagner-Storch and Palmer, 2003; Beerda et al., 2007). All of these factors should be considered when evaluating reported milk constituent values. Due to the labor-intensive nature of our study, we were only able to sample for a short duration during the summer. Further studies are necessary to study the seasonal patterns of early lactation constituents. Additionally, as shown in Teng et al. (2021), cows have varying milk composition depending on whether they are milked during the day or at night. Seely et al. (2022) also reported diurnal fluctuations in milk. Thus, we ensured consistency in sampling time relative to feed delivery so that constituents were recorded at the same time within the diurnal pattern, but it is possible that milk samples taken at other times of the day might show different patterns.

Another important limitation is that of the FTIR models. Although these PLS models have been created to analyze milk across most of a lactation, they were not designed to analyze constituents during the transition from colostrum to milk. Research by Sats et al. (2022) indicated that the fourth milking of a postpartum cow is colostrum transition milk, and all prior milkings are colostrum. Because the study farm milked 3 times a day, a cow could still be producing transition milk on 2 DIM, depending on when she calved. To account for this potential issue, we excluded milk samples from 0, 1, and 2 DIM, along with those samples where amounts (g/100 g) of de novo, mixed, and preformed FA summed to >99% of total FA. Another potential confounder we did not fully address was the potential influence of SCC on milk constituents. Although we excluded cow and milk samples from the study at the time of clinical mastitis diagnosis, SCC might have been increased in the days preceding the diagnosis. The implications of this increase in SCC on milk constituents should be further investigated in future studies.

To capture the daily changes in early lactation milk constituents more accurately across the first 10 DIM, we minimized confounding factors through the exclusion of transitional milk samples, the collection of milk samples at a consistent time each day, and the analysis of differences in constituents while controlling for parity group and milk yield per milking. Moving forward, with an increased understanding of parity influences on milk constituents in early lactation, we can begin to identify cows that deviate from the mean. Although there is inter-cow variation, daily testing of cows might be a powerful tool to identify within-cow changes indicative of maladaptation to the challenges of early lactation, and further investigation of within-cow changes is warranted.

## CONCLUSIONS

From the daily sampling of 1,024 Holstein cows, we described FTIR-estimated milk constituent concentrations for different early lactation parity groups on a single dairy farm. Our results suggest that parity is an important factor to consider when examining estimated milk constituents in early lactation to identify between-cow differences, and future studies should consider either a stratified parity approach or inclusion of parity as a covariate in explanatory models. Supplementary research on additional farms will help improve the external validity of reported constituent averages. With established milk constituent concentrations, daily continuous monitoring of milk offers the possibility to detect cows’ deviation from normal and provide additional support. More research is necessary to develop relevant models and test their accuracy.

## Figures and Tables

**Figure 3.**
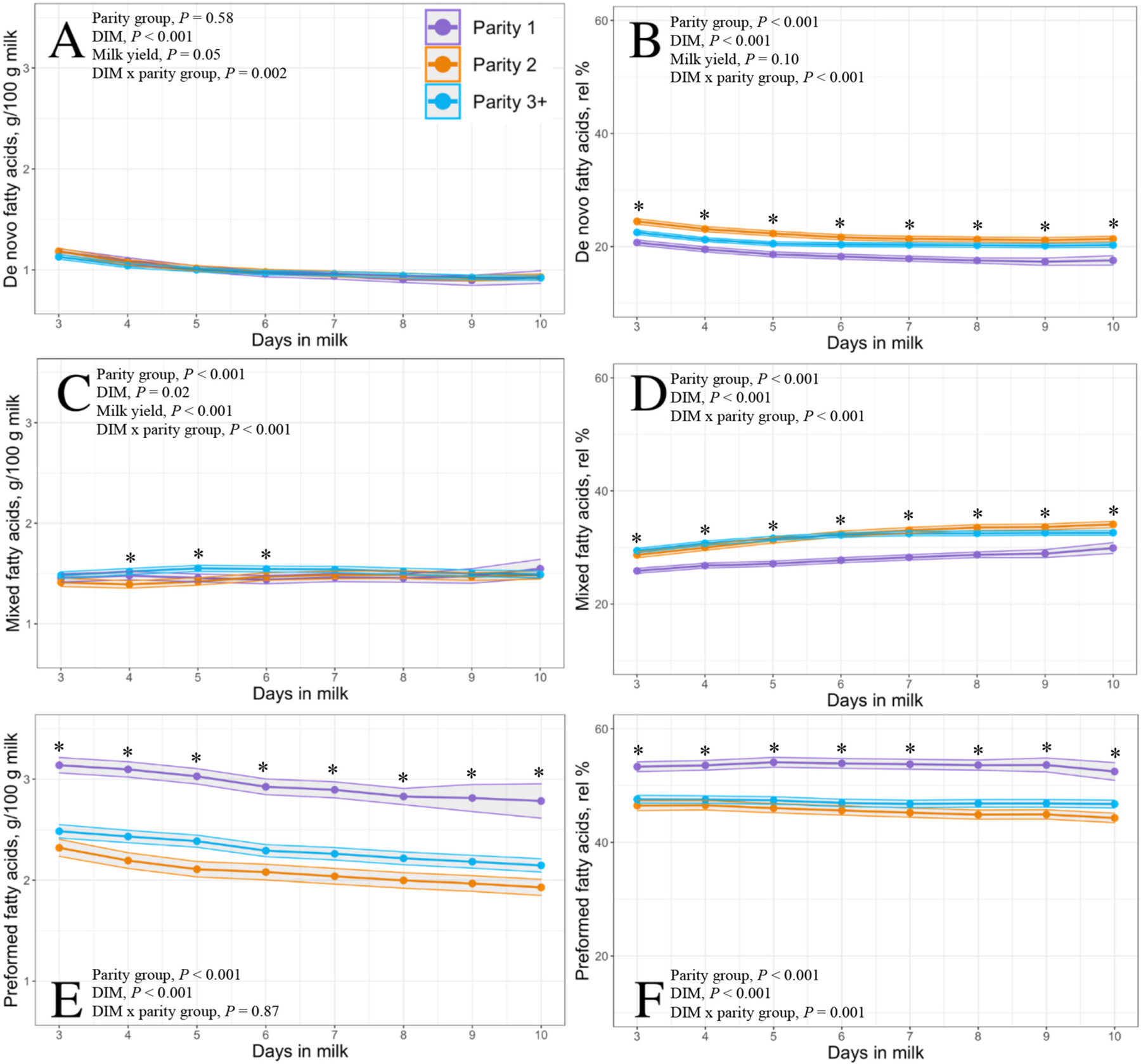
Final model LSM and 95% CI (shaded) of Fourier-transform infrared predicted grams of de novo (A), mixed (C), and preformed (E) fatty acids per 100 g of milk and respective relative percentage (rel %) of total fatty acids (B, D, F, respectively) from 3 through 10 DIM in 1,024 Holstein cows from a single dairy in New York. Parity groups are represented as parity 1 (purple; n = 306), parity 2 (orange; n = 274), and parity ≥3 (blue; n = 444). Asterisks represent a parity group difference at *P* < 0.05 at each respective DIM.

**Figure 4.**
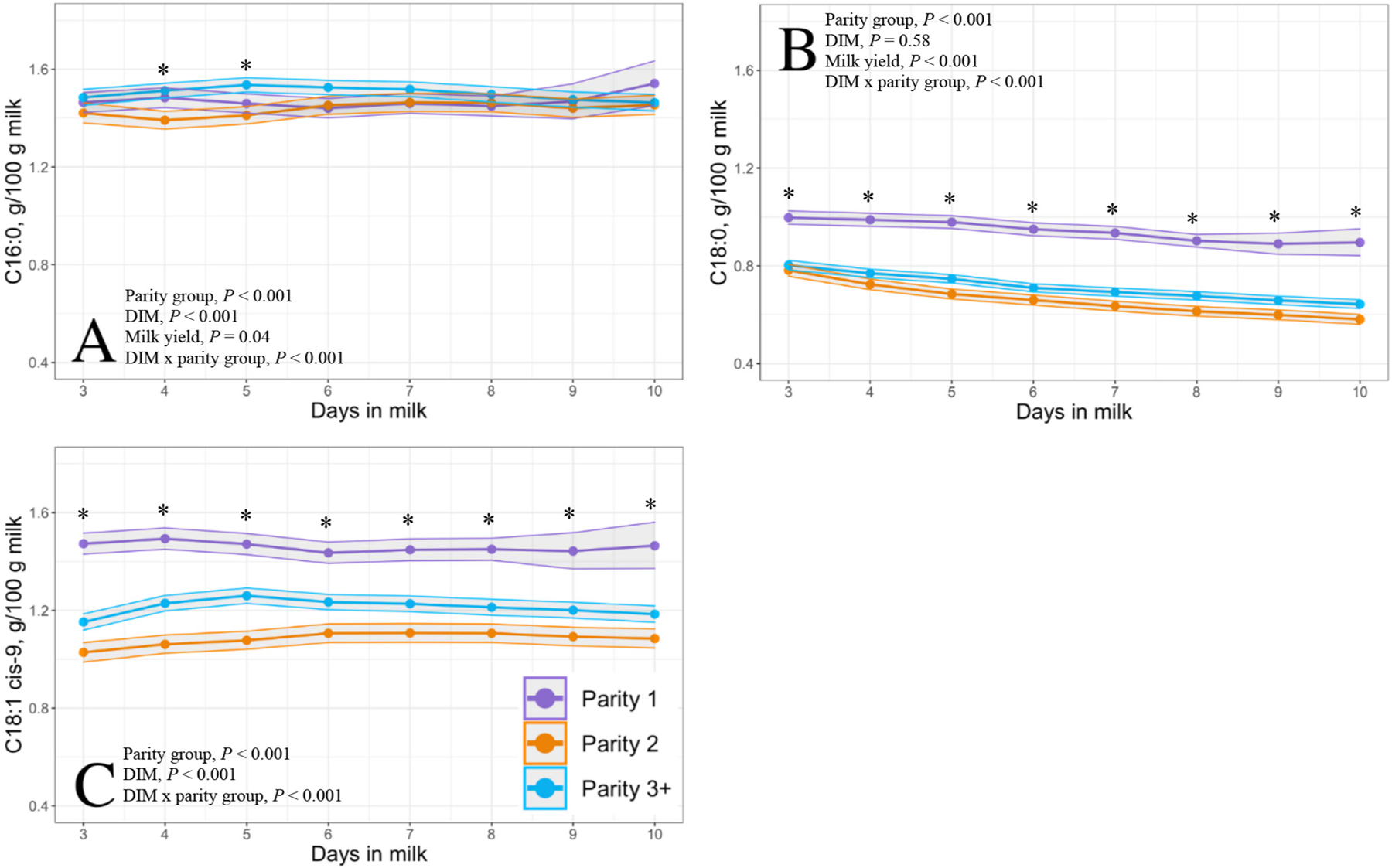
Final model LSM and 95% CI (shaded) of Fourier-transform infrared predicted grams of fatty acids C16:0 (A), C18:0 (B), and C18:1 *cis*-9 (C) per 100 g of milk from 3 through 10 DIM in 1,024 Holstein cows from a single dairy in New York. Parity groups are represented as parity 1 (purple; n = 306), parity 2 (orange; n = 274), and parity ≥3 (blue; n = 444). Asterisks represent a parity group difference at *P* < 0.05 at each respective DIM.

**Figure 5.**
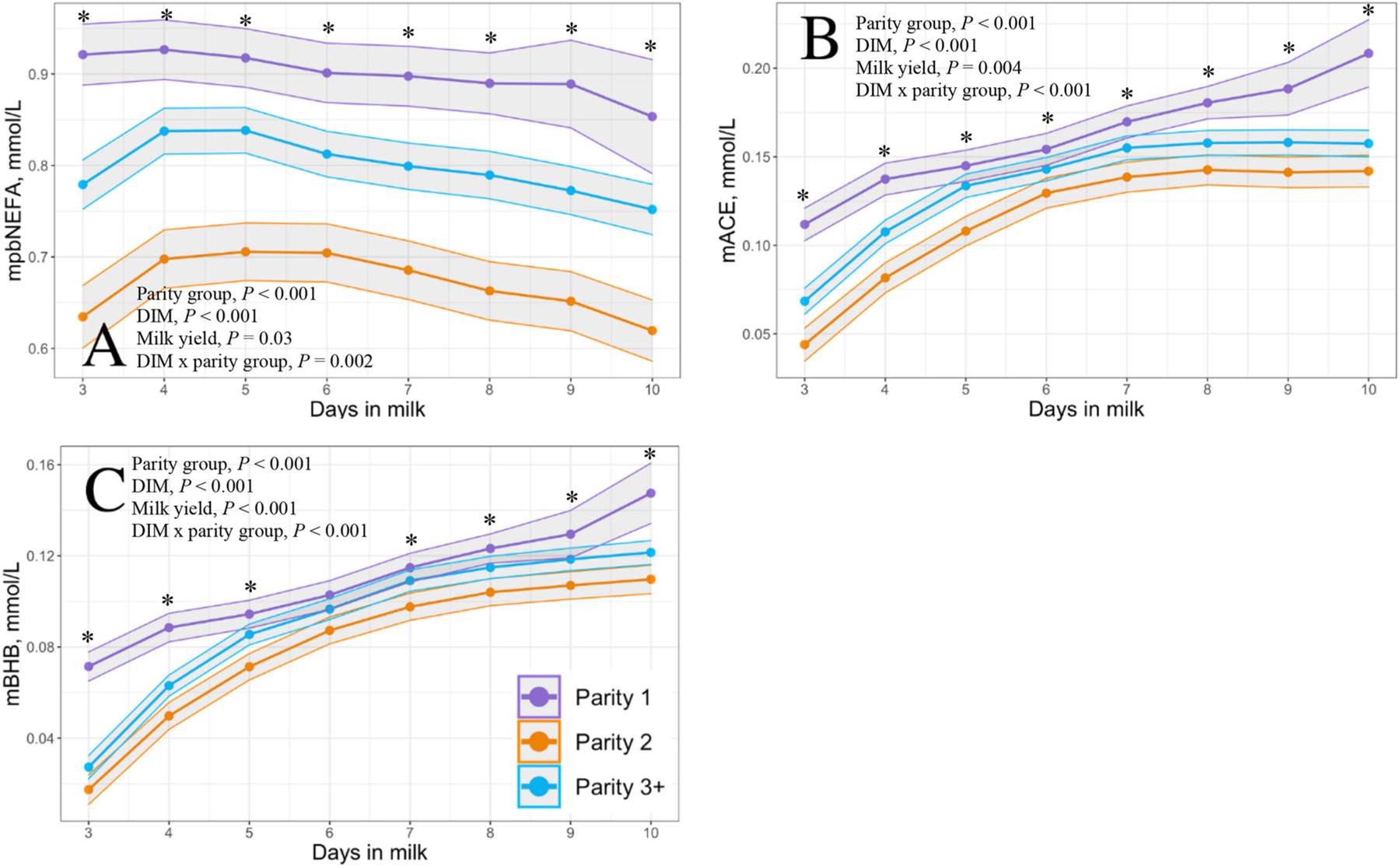
Final model LSM and 95% CI (shaded) of Fourier-transform infrared milk predicted blood nonesterified fatty acids (mpbNEFA) (A), milk acetone (mACE) (B), and milk BHB (mBHB) (C) from 3 through 10 DIM in 1,024 Holstein cows from a single dairy in New York. Parity groups are represented as parity 1 (purple; n = 306), parity 2 (orange; n = 274), and parity ≥3 (blue; n = 444). Asterisks represent a parity group difference at *P* < 0.05 at each respective DIM.

**Table 1.** Formulated early lactation diet ingredient and nutrient composition (% of DM unless otherwise noted; mean ± SD), calculated from 5 different diet estimation time points from June to August 2021, on a single commercial dairy farm in Cayuga County, New York

Item	Value
Ingredient	
Wheat straw	2.1 ± 0.2
Corn silage	40.0 ± 0.3
Haylage	18.8 ± 0.7
Whey blend	2.7 ± 0.0
Concentrates	36.4 ± 0.6
Nutrient composition	
DM	45.0 ± 0.3
NE_L_, Mcal/kg	1.7 ± 0.0
CP	17.7 ± 0.2
aNDF^1^	30.6 ± 0.3
Starch	23.8 ± 0.1
Ether extract	4.4 ± 0.1
Ca	0.9 ± 0.0
P	0.4 ± 0.0
Mg	0.3 ± 0.0
K	1.5 ± 0.0
S	0.3 ± 0.0
Na	0.4 ± 0.0
Cl	0.6 ± 0.0

1 aNDF = amylase-corrected neutral detergent fiber.

**Table 2.** Incidence (%) of early lactation diseases and culling (sold or died)^1^ within 30 DIM for all cows (n = 1,116) enrolled into the study, before placement of exclusion criteria, on a single commercial dairy farm in Cayuga County, New York, milking approximately 4,400 cows 3 times a day on a 100-stall rotary parlor

Disease/culling	Parity 1 (n = 349)	Parity 2 (n = 285)	Parity ≥3 (n = 482)
Milk fever^2^	0	1	5
Ketosis^3^	27	7	29
Metritis^4^	33	10	14
Displaced abomasum^5^	2	0	4
Mastitis^6^	28	24	23
Sold/died	14	4	10

1 Farm-diagnosed disease events extracted from farm computer records.

2 Diagnosed as a recumbent cow between 0 and 2 DIM.

3 Blood BHB concentrations ≥1.2 mmol/L on a cow-side meter test.

4 Systemic illness with a rectal temperature ≥39.5°C and reddish-brown uterine discharge.

5 A “ping” sound heard during simultaneous auscultation and percussion in a line from the tuber coxae to the olecranon.

6 Detection of a hot swollen quarter, presence of abnormal milk, or both.

**Table 3.** Final model milk yield per milking and estimated milk constituent concentrations (LSM, with 95% CI in parentheses) by parity group for 1,024 Holstein cows sampled from 3 through 10 DIM on a single commercial dairy farm in Cayuga County, New York

				*P*-value^1^			
Constituent	Parity 1	Parity 2	Parity 3+	Parity	DIM	Milk yield	DIM × parity
Milk yield, kg/milking	8.67^A^ (8.40, 8.95)	13.34^B^ (13.08, 13.61)	13.35^B^ (13.14, 13.56)	<0.001	<0.001	—	<0.001
Anhydrous lactose, % (wt/wt)	4.44^B^ (4.43, 4.46)	4.44^B^ (4.43, 4.46)	4.42^A^ (4.40, 4.43)	0.008	<0.001	<0.001	0.75
True protein, % (wt/wt)	3.48^A^ (3.45, 3.52)	3.77^B^ (3.74, 3.81)	3.82^C^ (3.80, 3.85)	<0.001	<0.001	<0.001	<0.001
Fat, % (wt/wt)	5.49^C^ (5.40, 5.59)	4.71^A^ (4.64, 4.77)	4.96^B^ (4.91, 5.02)	<0.001	<0.001	0.009	0.18
MUN, mg/100 g of milk	8.64^A^ (8.31, 8.97)	10.86^B^ (10.54, 11.18)	11.03^B^ (10.77, 11.28)	<0.001	<0.001	<0.001	<0.001
De novo FA,^2^ g/100 g of milk	0.99 (0.97, 1.01)	1.00 (0.98, 1.02)	0.99 (0.97, 1.00)	0.58	<0.001	0.05	0.002
Mixed FA, g/100 g of milk	1.47^A^ (1.44, 1.50)	1.45^A^ (1.43, 1.48)	1.52^B^ (1.50, 1.54)	<0.001	0.02	<0.001	<0.001
Preformed FA, g/100 g of milk	2.94^C^ (2.87, 3.00)	2.08^A^ (2.02, 2.14)	2.30^B^ (2.25, 2.35)	<0.001	<0.001	—	0.87
De novo FA, rel%^3^	18.42^A^ (18.01, 18.83)	22.10^C^ (21.68, 22.48)	20.69^B^ (20.37, 21.00)	<0.001	<0.001	0.10	<0.001
Mixed FA, rel%	27.89^A^ (27.49, 28.30)	32.04^B^ (31.61, 32.48)	31.75^B^ (31.41, 32.09)	<0.001	<0.001	—	<0.001
Preformed FA, rel%	53.54^C^ (52.75, 54.33)	45.49^A^ (44.75, 46.24)	47.09^B^ (46.49, 47.68)	<0.001	<0.001	—	0.001
C16:0, g/100 g of milk	1.47^AB^ (1.44, 1.50)	1.44^A^ (1.41, 1.46)	1.50^B^ (1.48, 1.52)	<0.001	0.58	<0.001	<0.001
C18:0, g/100 g of milk	0.94^C^ (0.92, 0.96)	0.66^A^ (0.64, 0.67)	0.71^B^ (0.70, 0.72)	<0.001	<0.001	0.04	<0.001
C18:1 *cis-9,* g/100 g of milk	1.46^C^ (1.42, 1.50)	1.08^A^ (1.05, 1.11)	1.21^B^ (1.19, 1.24)	<0.001	<0.001	—	<0.001
mpbNEFA,^4^ mmol/L	0.90^C^ (0.87, 0.93)	0.67^A^ (0.64, 0.70)	0.80^B^ (0.78, 0.82)	<0.001	<0.001	0.03	0.002
mBHB,^5^ mmol/L	0.11^C^ (0.10, 0.11)	0.08^A^ (0.08, 0.09)	0.09^B^ (0.09, 0.10)	<0.001	<0.001	<0.001	<0.001
mACE,^6^ mmol/L	0.16^C^ (0.15, 0.17)	0.12^A^ (0.11, 0.12)	0.14^B^ (0.13, 0.14)	<0.001	<0.001	0.004	<0.001

A–C Values within a row with different superscripts represent differences (*P* < 0.05) in means separated by Tukey’s studentized adjustments to account for multiple comparisons.

1 Associated type III *P-*values obtained from the generalized linear mixed models.

2 Fatty acid.

3 Relative percentage = percentage of total FA.

4 Milk-predicted blood nonesterified FA.

5 Milk BHB.

6 Milk acetone.
